# The importance of disease prevalence in clinical decision making: a real practice study on COVID-19 antigen test in Curacao

**DOI:** 10.1016/j.bjid.2022.102389

**Published:** 2022-07-22

**Authors:** Erlangga Yusuf, Liane Virginia-Cova, Lisette B Provacia, Jeanne Koeijers, Vanessa Brown

**Affiliations:** a Curacao Medical Center, Willemstad, Curacao; b Analytisch Diagnostisch Centrum NV, Willemstad, Curacao; c Erasmus University Medical Center, Rotterdam, the Netherlands

**Keywords:** COVID-19, Rapid antigen test, Performance, Sensitivity, Positive predictive value

## Abstract

The performance of a test can be suboptimal, but in appropriate setting such a test is still useful for clinical decision making. We investigated the role of Antigen Rapid Diagnostic Test (Ag-RDT) for clinical decision making in an Emergency Department (ED) in Curacao during peak of COVID-19 pandemic. Ag-RDT was performed in the naso- and oropharynx-swabs from patients with respiratory insufficiency presented to the ED. Ag-RDT was performed in 153 patients, of which 64 (41.8%) showed positive results. Comparing Ag-RDT results with molecular tests, its sensitivity was 68.8% (95% CI 57.4 to 78.7), and specificity of 94.6% (95% CI 84.9 to 98.9). The positive and negative predictive value were 95.1% (95% CI 86.5 to 98.3) and 66.3 (95% CI 58.6 to 73.3), respectively. All patients with Ag-RDT positive test were admitted to the cohorted COVD-19 department of the hospital. By using Ag-RDT, 35.9% of rapid PCR tests (that are more costly and laborious to perform) could be avoided at cost of 5.8% patients with false positive result. In conclusion, in real practice, disease prevalence is as important as test's performance for clinical decision making. The conclusion may also be applicable for other diagnostic tests than COVID-19 diagnostic.

## Introduction

As the prevalence of a disease increases, positive predictive value (PPV) of a test will also increase.^1^ Prevalence is thus an important factor to take into account in choosing a diagnostic test, but it is often ignored since one tends to focus only on the performance (sensitivity and specificity) of the test itself.

In COVID-19 diagnostics, the performance of Antigen Rapid Diagnostic Test (Ag-RDT) is often suboptimal. The average sensitivity in the first week after symptom onset is 78.3% (95% CI 71.1% to 84.1%), and the specificity of 99.6% (95% CI 99.0% to 99.8% (2). While it is not as performant as molecular tests, the gold standard in COVID-19 diagnosis, Ag-RDT has several advantages. It is readily available, cheap, and can be performed rapidly in the proximity of the patient.^2^^,^^3^

On March 30^th^, 2021, Curacao, an island in the Dutch Caribbean with 160,000 inhabitants, registered 506 new positive COVID-19 cases. At that moment, this number per capita was the highest in the world. A proportion of these positive cases could be expected to visit the Emergency Department of the only COVID-19 hospital in Curacao. The anticipated number of patients would largely exceed the number of rapid COVID-19 molecular test available in the hospital. This rapid molecular test was used to triage the patients into COVID-19 or non-COVID-19 department. Due to global competition, the supply of cartridges for molecular test could not be increased within a short time.

As a result of this situation, we implemented Ag-RDT as a screening test despite of its suboptimal performance. We hypothesize that due to the high disease prevalence, the PPV would also increase, and Ag-RDT would be a useful resource in this setting. Therefore, the aim of this study was to investigate the performance and the usefulness of the Ag-RDT test in a resource limited setting at the peak of the pandemic.

## Material and methods

### Study setting

This was a retrospective observational study performed at Curacao Medical Center, a hospital with 270 beds, including 10 intensive care unit (ICU) and 6 medium care beds. During the peak of COVID-19 pandemics, the ICU capacity was increased to 52 beds. The medical laboratory service of this hospital is provided by a semi-governmental laboratory that performed 600 COVID-19 PCR tests a day during the peak week of pandemic (the normal capacity was 100 to 200 tests a day). We included laboratory data of patients who visited the Emergency Department in the first two weeks of April 2021 (i.e. two weeks following peak of number of positive tests in Curacao on March 30^th^, 2021).

In the week previous to March 30^th^, 2021, there was on average one death per week. The peak weekly average death rate was reached in the week starting on April 18^th^, 2021, when on average three deaths per week were registered. On March 30^th^, only 10.2% of the population has received at least dose of COVID-19 vaccination, and 3.1% was fully vaccinated.

This study used retrospective data collected in routine practice and the tests were performed for clinical purposes, therefore it was not subjected to institutional review board approval according to the Dutch law (‘niet WMO plichtig’).

### Tests and test algorithm

The Ag-RDT used in this study was the Novel Coronavirus (SARS_Cov-2) Antigen Rapid Test (Hangzhou Realy Tech). Prior to its use in our hospital, no published data were available regarding the performance this Ag-RDT. Therefore, we performed a verification study in 15 nasopharyngeal swabs by comparing the Ag-RDT results with results from real-time PCR targeting the E-gene (cobas® SARS-CoV-2 Test, Roche Diagnostics). The sensitivity of the Ag-RDT was 50% when all positive samples were taken into account, and 100% when only positive samples with Ct-values between 15 and 25 (*n* = 5) were included. The specificity of the test was 100%.

In the Emergency Department, a two-tiered test algorithm was performed. The patients presented at the Emergency Department with clinical symptoms (fever, cough, shortness of breath, oxygen saturation <94%) and signs (pulmonary rales, infiltration on the chest X-ray) severe enough for a hospital admission, were screened using Ag-RDT. Positive Ag-RDT was confirmed using light-cycler PCR as above on the next day, but when Ag-RDT showed negative result, a rapid PCR test (Xpert® Xpress SARS-CoV-2, Cepheid) was immediately performed as per manufacturer instruction.

### Statistical analysis

We created a contingency table and calculated the sensitivity, specificity, PPV and negative predictive value (NPV) of the Ag-RDT (with their 95% confidence intervals (95% CI)) by comparing the results of this Ag-RDT test with those of molecular tests. Using this table, we also calculated the number of molecular tests that could be spared using the two-tiered algorithm. The prevalence of COVID-19 was calculating as the proportion of positive PCR or positive Ag-RDT tests among the tested individuals during the study period.

We also performed a sensitivity analysis by calculating the performance of the test only during the long Easter weekend (between April 2^nd^ and April 5^th^, 2021) that directly followed the peak of positive COVID-19 tests in the population (March 30^th^, 2021). This was performed because the patient characteristics (more or less eager to visit the Emergency Department) or the personnel's (more or less experience) might differ from those during the working week. For this sensitivity analysis, we calculated the prevalence as the proportion of these patients testing positive for rapid PCR test in the week prior to March 30^th^, 2021.

Analyses were performed using IBM SPSS Statistics for Windows version 26.0 (IBM Corp., Armonk, NY, USA).

## Results

### Performance of the Ag-RDT algorithm in the real practice

During the study period, 153 patients were included, and 64 (41.8%) had positive Ag-RDT test results. PCR data were available from 132 patients. In 15 patients with negative Ag-RDT results, PCR was not performed while this supposed to be performed according to the algorithm, and in six patients with positive Ag-RDT, no confirmation PCR was performed. The COVID-19 prevalence in this setting was 60.6%.

The contingency Table on the performance of Ag-RDT in comparison to the PCR is shown in Table 1. All positive PCR tests had Ct-value of < 35 cycli. The sensitivity of the Ag-RDT was 68.8% (95% CI 57.4 to 78.7), and specificity of 94.6% (95%C I 84.9 to 98.9). In this setting, the PPV was 95.1% (95% CI 86.5 to 98.3) and the NPV was 66.3 (95% CI 58.6 to 73.3).

**Table 1:** Contingency table comparing COVID-19 Ag-RDT with COVID-19 PCR tests in 132 patients presenting with clinical symptoms and signs at the Emergency Department during the study period.

		**PCR**	
		Positive	Negative
**Ag-RDT**	Positive	55	3
	Negative	25	49

By using Ag-RDT, 55/153 (35.9%) of rapid PCR tests could be avoided at cost the of 5.8% patients with false positive result.

### Sensitivity analysis

Including patients presented at the Emergency Department at the Easter weekend only (*n* = 59), 28 (47.4%) of the Ag-RDT tests was positive. The sensitivity of the Ag-RDT was 65.8% (95% CI 48.7 to 80.4), and specificity of 91.7% (95% CI 61.5 to 99.8). Using the prevalence from the week prior (62.9%, (42/61 patients were tested positive using PCR)), the PPV was 93.1% (95% CI 66.9 to 98.9) and the NPV was 61.3 (95% CI 49.6 to 71.7). Rapid PCR test could be spared in 28/59 (47.4%), at cost of 1/59 (1.7%) patient with false positive result.

## Discussion

We showed that in high disease prevalence setting, the PPV of a test is also high, and a test with suboptimal performance can still have an important role in clinical decision making. Moreover, regarding COVID-19 diagnostic, the use of Ag-RDT use has led to significant sparing of rapid PCR test in our hospital.

Our present study provides support to a theoretical framework on positioning Ag-RDT in the real practice.^3^ This theoretical framework calculated that when the likelihood to be tested positive (prevalence) is 25–50%, an Ag-RDTs with sensitivity as low as 80% (and 97% specificity), would have positive predictive value of 90–96%, provided that the test is used within seven days after symptom onset.^3^ This framework also posed that Ag-RDT could be used in a certain setting, such as quick triaging and when demand for COVID-19 rapid molecular tests exceeds molecular testing capacity. These conditions were applicable in our setting. Another benefit of using Ag-RDT is the cost saving. The cost of Ag-RDT was estimated at least 8-fold cheaper than commercial PCR test.

This study helps to remind clinicians and other stakeholders in the diagnostic process, that prevalence should be considered when implementing a diagnostic test, and not only based on sensitivity and specificity of the test. This holds true not only for COVID-19 diagnostic test, but also for other clinical diagnostic tests. While it is desirable to have a test with high sensitivity and specificity, these two parameters are of limited use when decision needs to be made in estimating the probability of a disease in an individual.^4^ In this context, PPVs and NPVs are more appropriate. PPV is the probability that an individual with a positive test result has indeed the disease. PPV and NPV, but not sensitivity or specificity, are influenced by the prevalence of disease in the population that is being tested.

The World Health Organization (WHO) recommended the minimum sensitivity as 80% for a COVID-19 Ag-RDT (compared to a molecular test).^5^ Several studies on various Ag-RDT use in the Emergency Departments have been published.^6^, ^7^, ^8^, ^9^, ^10^, ^11^ Mostly, studies performed Ag-RDT in all type of patients attending the Emergency Department, and not necessarily in patients with high suspicion of respiratory insufficiency due to COVID-19. Also, these studies often did not report the prevalence, so the PPV could not be calculated. In a low prevalence setting in an Emergency Department, the role of Ag-RDT would be limited, as shown in a study where in 116 patients screened upon admission in a 250-bed community hospital in Switzerland. In this study, Ag-RDT detected 2/7 rapid PCR positive patients and delivered two false positive results.^12^

We also noticed the discrepancy between data from the manufacturer, the verification study and the real practice. The manufacturer reported the sensitivity of 96.2%, and the specificity of 100.0% (package insert). This performance was confirmed in our verification study using historical samples only in samples with PCR results with Ct-value of < 25. In real practice, the sensitivity was much lower. The discrepancy could be explained due to sampling error and inappropriate handling of the test. It is therefore important to take into account these possible differences, and to assess the performance of the Ag-RDTs or any other diagnostic tests in routine practice, rather than the performance reported by the manufacturer or verification study.

The strengths of this study are the use homogenous population in a real setting. Several limitations of this study should be acknowledged such as missing molecular data that reflected the daily practice.

In conclusion, we showed that Ag-RDT, despite its suboptimal performance, can help clinical decision making in high prevalence setting.

## Conflicts of interest

The authors declare no conflicts of interest.
